# Ipsilateral Facial Hyperhidrosis in a Patient With Squamous Cell Carcinoma of the Lung

**DOI:** 10.7759/cureus.24832

**Published:** 2022-05-08

**Authors:** Tamara Lynne B Aqui, Neil K Patel, Yolanda Zhang, Scott Kubomoto

**Affiliations:** 1 Internal Medicine, Riverside Community Hospital, Riverside, USA; 2 Internal Medicine, University of California, Riverside School of Medicine, Riverside, USA

**Keywords:** ipsilateral, hemifacial, lung, squamous cell carcinoma, hyperhydrosis, cancer

## Abstract

While anhidrosis in Horner’s Syndrome is a well-documented result of apical lung malignancies impinging on the sympathetic pathway traveling through the upper lobe of the lung, its opposite effect, hyperhidrosis, is a seldom reported consequence. Hyperhidrosis occurs as a result of irritation of the sympathetic nervous system near the superior cervical ganglion. In this report, we examine a patient with known Stage IIIB squamous cell carcinoma of the lung presenting with right hemifacial hyperhidrosis, dyspnea, and right upper extremity swelling. Computed tomography angiography (CTA) of the chest re-demonstrated the intrathoracic neoplasm encroaching on his mediastinum. During admission, the patient had his first cycle with carboplatin and paclitaxel. His hyperhidrosis, as well as dyspnea and swelling improved post-treatment, and the patient was stable for discharge to follow up outpatient with oncology and radiation oncology to continue further treatment. As hemifacial hyperhidrosis is rarely reported, it becomes important to recognize this as a likely indicator of mediastinal invasion from malignancy.

## Introduction

In the United States, lung cancer ranks second in incidence rates among both sexes. Five-year survival rate peaks at 56% when diagnosed at a localized stage; however, only 16% of diagnoses are made at this stage, making early recognition of symptoms vital to receiving prompt treatment [1]. The triad of ptosis, miosis, and anhidrosis found in Horner’s Syndrome is often seen in lung cancer, with anhidrosis being caused by the interruption of the sympathetic chain [2]. Hyperhidrosis is a rare phenomenon that has been documented in patients with lung malignancies in a handful of case reports. We discuss the case of a man with known squamous cell carcinoma of the lung presenting with dyspnea, facial swelling, and profuse diaphoresis on one side of the face, which improved after treatment initiation.

## Case presentation

**Figure 1:**
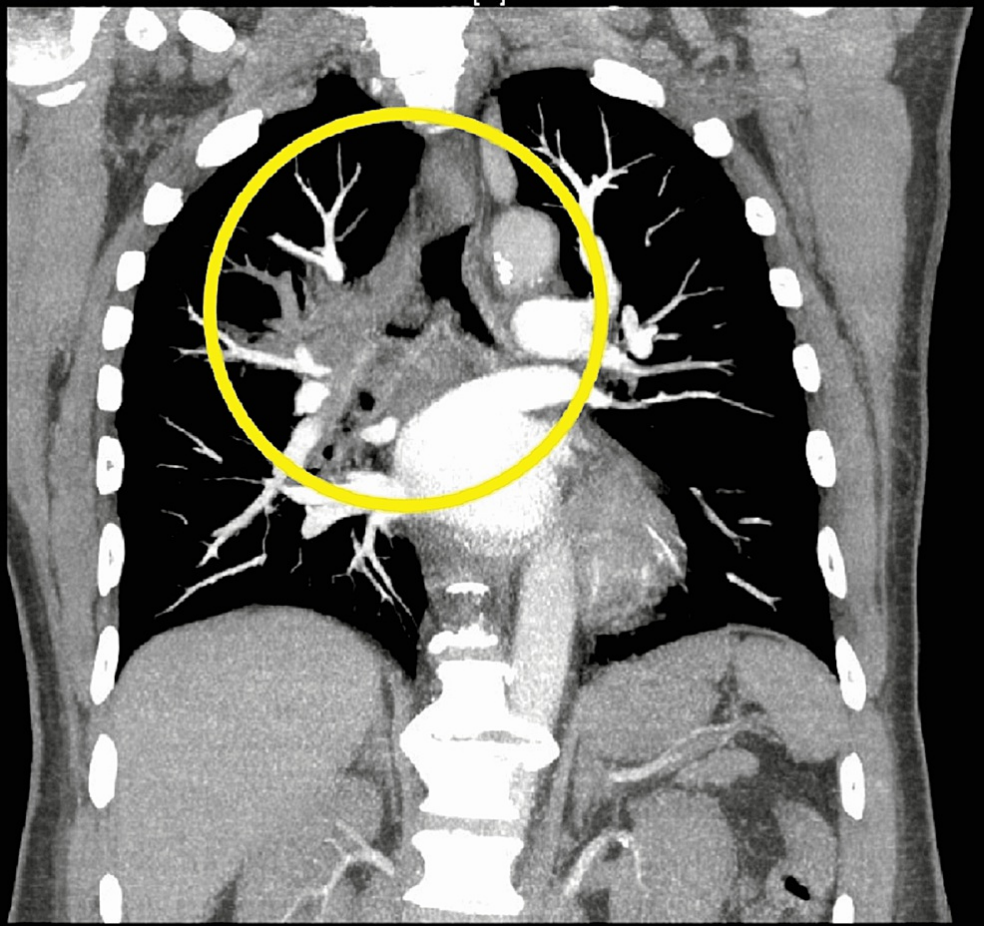
Computed Tomography Angiography of the Chest Encircled in yellow is a right hilar mass infiltrating the mediastinum measuring approximately 4.8 cm anteroposterior by 7.3 cm transverse dimensions, causing narrowing of the right bronchus and right pulmonary vasculature

A 60-year-old man with known Stage IIIB squamous cell carcinoma of the right lung and heart failure with reduced ejection fraction presented to the emergency room with dyspnea, right upper extremity swelling, and right hemifacial diaphoresis. The onset of dyspnea and diaphoresis was associated with his cancer diagnosis two months prior and have been worsening. Right upper extremity swelling started with the placement of his chest port for chemotherapy one month prior, but he had not started chemotherapy due to relocation in order to be closer to family. Beads of sweat were prominently visible on the right half of his face while in the emergency department, while the left side remained dry. Vital signs were stable, and the patient required two liters of oxygen via nasal cannula with a saturation of around 95%. Computed tomography angiography (CTA) was performed, which demonstrated the large infiltrative mass centered in the right hilum consistent with the known malignancy, extending into the mediastinum and right upper lobe (Figure 1). The mass caused severe narrowing of the superior vena cava, right upper lobe pulmonary artery, and right upper lobe bronchus. Surgical intervention was deferred, as the patient only had mild superior vena cava symptoms, and the mass was potentially amenable to chemotherapy. Unilateral hyperhidrosis decreased while he was inpatient, with the diaphoresis only being noticeable on the right via touch. He was given one round of chemotherapy with carboplatin and paclitaxel during his admission and discharged on room air to continue his chemotherapy outpatient. 

## Discussion

Direct invasion of the sympathetic chain from a superior sulcus tumor causes an interruption in nerve supply, resulting in the classical effects of Horner syndrome. The ipsilateral anhidrosis seen in Horner comes from the disruption of the second-order neurons involving the face [2]. First-order neurons in the hypothalamus sense change primarily in temperature and terminate in the spinal cord around the C8-T2 levels [2]. Fibers from the second-order neuron then exit out the T1 nerve root and traverse the paravertebral sympathetic chain and stellate ganglion to terminate in the superior cervical ganglion. Post-ganglionic fibers course along the external carotid artery and end in the sweat glands of the face [3]. 

Instead, the ipsilateral hyperhidrosis experienced by our patient is most likely the result of a localized irritation of the sympathetic chain around the cervical sympathetic ganglion instead of interruption [4]. This was postulated by Hepper et al. to eventually lead to the symptoms seen in Horner syndrome as the tumor invades and begins to interrupt signaling [5]. While sweating happens in response to temperature changes, emotional state, and physical activity, the association between hyperhidrosis and malignancy is usually independent and occurs randomly [6]. At the time of presentation, the patient was at rest in a cool room when perspiration was noted. Radiotherapy has been used in some cases for symptom relief with varying degrees of success; however, the patient did not receive any treatment specifically for his hyperhidrosis and did not appear to adversely affect his quality of life [7,8].

## Conclusions

Lung cancer remains a major source of cancer mortality in the United States. It is important to recognize that ipsilateral hyperhidrosis can be a presenting symptom of an intrathoracic malignancy due to the irritation it can cause to the sympathetic chain. While chemotherapy and radiotherapy can help alleviate symptoms, overall prompt identification and treatment of the malignancy is the main goal.
